# Parental mood during pregnancy and post-natally is associated with offspring risk of Tourette syndrome or chronic tics: prospective data from the Avon Longitudinal Study of Parents and Children (ALSPAC)

**DOI:** 10.1007/s00787-015-0742-0

**Published:** 2015-07-15

**Authors:** Y. Ben-Shlomo, J. M. Scharf, L. L. Miller, C. A. Mathews

**Affiliations:** School of Social and Community Medicine, University of Bristol, 39 Whatley Road, Bristol, BS8 2PS UK; Psychiatric and Neurodevelopmental Genetics Unit, Center for Human Genetics Research, Massachusetts General Hospital, Boston, MA USA; Departments of Psychiatry and Neurology, Massachusetts General Hospital, Boston, MA USA; Division of Cognitive and Behavioral Neurology, Department of Neurology, Brigham and Women’s Hospital, Boston, MA USA; Program for Genetics and Epidemiology of Neuropsychiatric Symptoms, Department of Psychiatry, University of California, San Francisco, San Francisco, CA USA

**Keywords:** Tourette syndrome, Anxiety, Depression, Cohort studies, Risk factors, ALSPAC

## Abstract

Little is known about risk factors for Tourette syndrome (TS) and chronic tic disorders (CT) but maternal psychological morbidity in pregnancy may be associated with TS/CT. We examined whether pre- and post-natal parental anxiety and/or depression are associated with risk of TS/CT in the Avon Longitudinal Study of Parents and Children. We compared self-reported anxiety and depression measures collected prospectively at four time points (18 and 32 weeks prenatally, and 8 weeks and 8 months post-natally) among parents of children who subsequently met criteria for TS/CT at 13 years of age as compared to other children from the cohort. We adjusted for various socioeconomic measures and tested both for time period-specific exposure and chronic exposure using multivariable logistic regression models. 122 children had TS/CT (50 TS, 72 CT) and 5968 children had no tics. In crude analyses, both pre- and post-natal maternal anxiety and depression, but only post-natal paternal depression at 8 months, showed associations with TS/CT. In the final, adjusted multivariable models, chronic maternal anxiety (odds ratio 2.17, 95 % CI 1.23, 3.84, *p* = 0.007) and pre-natal maternal depression (odds ratio 1.86, 95 % CI 1.02, 3.39, *p* = 0.04) showed associations with TS/CT though the latter was consistent with chance (*p* = 0.07) after adjustment for past maternal depression. We find associations between maternal psychological morbidity pre- and post-natally and risk of future TS/CT in offspring. These associations may reflect either shared genetic susceptibility or a pre-natal exposure. Further work is required to see if these findings can be replicated in larger datasets.

**Electronic supplementary material:**

The online version of this article (doi:10.1007/s00787-015-0742-0) contains supplementary material, which is available to authorized users.

## Introduction

Remarkably little is known about risk factors for Tourette syndrome (TS) and other chronic tic disorders (CT). Although susceptibility to TS and CT is clearly genetic, at least in part, evidence from heritability studies suggests that between 25 and 50 % of risk may be due to environmental factors [1]. However, it is unclear which potential non-genetic factors are of relevance for TS/CT, and when in the life course such exposures may convey risk. For neurodevelopmental disorders such as TS/CT, it is plausible that exposures in the pre- and post-natal period may have disproportionate influence due to fetal or post-natal programming of the developing brain and neural circuitry. Over the last decade or more, chronic disease epidemiologists have increasingly adopted a life course epidemiological perspective, [2] as this provides a helpful conceptual framework to examine when potential environmental exposures may be of importance. Within this approach, there are a variety of potential causal models [3] that need to be considered when testing for the importance of specific exposures on disease causation. In particular, exposures could operate through a “critical period” effect (if exposures that occur only during this time frame contribute to disease) or a “sensitive period” effect (if exposure outside this postulated time window is also important but of lesser magnitude). Alternatively, exposures may simply accumulate over time, so that an earlier onset of exposure may appear to be detrimental due a greater total duration of exposure, analogous to cigarette pack-years for lung cancer [4].

There is increasing interest in the potential role of maternal psychological morbidity in pregnancy as a risk factor for neurological and psychiatric conditions in the offspring [5]. For example, in the Avon Longitudinal Study of Parents and Children (ALSPAC) cohort, pre-natal maternal anxiety (but not pre-natal maternal depression) was associated with children’s behavioral and emotional problems at age 4 [5]. Further, pre-natal maternal anxiety appeared to act in an additive way with post-natal maternal depression, potentially suggesting a complex interaction of biological and environmental contributors.

Maternal psychopathology may affect children’s risk of neuropsychiatric disorders in a variety of different pathways. Firstly, depressed mood during pregnancy may be a marker of chronic psychological morbidity (i.e., increased genetic liability) that predates pregnancy but is exacerbated by it. In this case, one would hypothesize that paternal genes should also be contributory to TS/CT risk, and thus one would expect to see similar associations between TS/CT and both maternal and paternal depression. Secondly, an association between maternal mood/anxiety and neuropsychiatric disorders in a child may reflect confounding from external circumstances that increases psychosocial stress acutely during pregnancy. In this scenario, there should also be similar associations between maternal and paternal symptoms and neurological and psychiatric conditions in the offspring, although these would be greatly attenuated after adjustment for socioeconomic factors. Thirdly, there may be specific effects of maternal mood/anxiety symptoms on the developing fetal brain. In this case, one would predict that any adverse effect would be seen with maternal but not paternal exposure.

To date, there have been only two studies examining the relationship between maternal psychological morbidity in pregnancy and chronic tic disorders, both retrospective in nature. One small study reported that the severity of maternal life events during pregnancy predicted tic severity among individuals with TS [6]. The second showed a trend toward increasing levels of severe maternal psychological stress during pregnancy in association with TS compared to controls [7]. However, socioeconomic status was not controlled for in either study. We have previously shown that lower socioeconomic status is also associated with a greater risk of TS and chronic tics (CT), [8] so it is unclear whether these prior associations merely reflect socioeconomic confounding, as suggested by a previous study of maternal and paternal depression and attention deficit problems [9]. To date, no prospective studies examining the relationship of maternal psychological stress and TS/CT have been published.

This study examines the association between maternal and paternal anxiety and depression and risk of TS/CT in the ALSPAC, a prospective pre-birth cohort with extensive parental and offspring data [10]. To our knowledge, this is the first prospective examination of the association between parental psychological morbidity and tic disorders. Although known to be highly heritable, little is still known about the specific risk factors for TS/CT, and even less about non-genetic risk factors. Therefore, this study represents an important contribution to our understanding of the etiology of this complex disorder. We predicted a priori that if maternal stress is a risk factor for TS/CT, then maternal reporting of anxiety would show a stronger relationship than paternal reporting of similar symptoms, and that the association would be stronger for pre- than for post-natal exposure, given the greater sensitivity of the fetal brain to any adverse exposure. This association should also be robust after adjustment for a wide range of socio-demographic and other peri-natal confounders.

## Materials and methods

The ALSPAC is an ongoing, prospective, population-based, pre-birth cohort study of all children born to 14,541 pregnant women resident in Avon, UK (representing 85 % of the eligible population) with expected dates of delivery between April 1st 1991 and December 31st 1992 [11, 12]. Of the initial 14,541 pregnancies, 14,472 had known birth outcomes, resulting in a total of 14,676 fetuses (195 twin births, 3 triplets and 1 quadruplet). There were 14,062 live births and 13,988 children were alive at 1 year. Ethical approval for the study was obtained from the ALSPAC Law and Ethics Committee and the Local Research Ethics Committees.

Self-administered maternal questionnaires began at the time of enrollment in early gestation and continued throughout pregnancy, with detailed questions regarding demographics, educational and vocational history, medical and family history, diet and life style exposures. Post-partum questionnaires were supplemented by obstetric and pediatric medical data from the two major maternity hospitals in the region. Post-natally, mothers completed questionnaires about themselves and their children’s development, environmental exposures and health outcomes approximately every 6 months from birth to age 7 and every year thereafter, with data available for 7152 children at ages 13–14. Partners also completed questionnaires yearly. Starting at age 6, children were invited to attend yearly clinics for direct clinical assessments (see ref [10] for more details concerning ALSPAC). The generalisability of the ALSPAC sample has been previously reported [12]. Briefly, children in Avon had parents with a similar racial distribution (5.1 vs. 6.4 % non-white), level of education (14.0 vs. 13.7 % with university degrees), and single parent household at age 5 (4 vs. 5 %) as the general UK population, although children in Avon were significantly less likely to have a father working in manual labor than children from the general UK population (51.6 vs. 65.1 %).

### Outcome measures

The primary outcome measures were diagnoses of TS or CT. The ALSPAC questionnaires contained screening questions for recurrent tics at eight time points over 9 years (from ages 1.5–10), as well as a more detailed set of questions about specific types of motor and vocal tics at age 13, allowing for the assignment of TS or CT diagnoses [13]. Details about the tic-related questions and the derivation of the TS and CT diagnoses used in this study are summarized in Scharf et al. [13], and complete questionnaire data are available at the ALSPAC website data dictionary: http://www.bristol.ac.uk/alspac/researchers/data-access/data-dictionary/. TS and CT diagnoses were assigned based on DSM-IV-TR criteria, and required the presence of both motor and vocal tics at age 13 at the “definite” or “probable” level, occurring daily or more than once a week, along with a positive endorsement of tics from at least one prior time point. Participants who had only repeated movements of the arms, hands, legs or feet or repeated words or phrases in the absence of a positive response to other tic questions were excluded from all case definitions to remove non-tic movements such as stereotypy or isolated echolalia. Individuals with intellectual disability (defined here as Verbal IQ ≤80) or autism based on medical and school record review or on neuropsychological testing were also excluded [13]. Sensitivity analyses, and examination of gender ratios and rates of obsessive–compulsive disorder (OCD) and attention deficit hyperactivity disorder (ADHD), which are highly comorbid with TS/CT, suggest that these definitions have face validity and represent true cases of TS and CT [13]. The final sample for this study consisted of 6140 children, including 50 with TS, 72 with CT (for a total of 122 with either TS or CT) and 5968 children with no tics. 70 % of TS cases were male, 20 % had OCD and 18 % had ADHD; in the combined TS/CT case sample, 71 % were male (*n* = 51), 14 % had OCD (*n* = 17), and 14 % had ADHD (*n* = 17) [13].

### Predictor variables and potential confounders

Maternal and paternal anxiety was measured using the self-rated Crown-Crisp index [14]. This validated measure produces a total ordinal score with higher values indicating greater anxiety. Data were collected from the mother on four occasions at around 18 and 32 weeks prenatally and 8 weeks and 8 months post-natally. The same questionnaire was given to the fathers at these time points, although fathers did not receive a 32 week questionnaire. Depressive symptoms were captured using the Edinburgh postnatal depression scale [15], which is a ten item self-reported measure with higher scores indicating greater depression. The Edinburgh postnatal depression scale has been shown to be valid pre-, peri- and post-natally. Depression data were collected for mothers and fathers at the same time points as the anxiety data. Given the possibility of non-linear relationships between parental symptom severity and offspring TS/CT, we examined anxiety and depression scores both as quantitative measures and as categorical variables, grouping them into tertiles given the limited number of TS and CT cases.

Potential confounders included socioeconomic status and a variety of pre- and post-natal exposures that have been associated with increased risk of TS/CT in this population [8, 16]. To control for socioeconomic status, we used a standardized composite measure that was derived from a variety of post-natal variables (housing tenure, private garden/yard use, access to a car, and financial difficulties) and grouped into tertiles. We have previously shown, using this measure, that lower SES is associated with increased risk of TS/CT in this sample [8]. In addition, we adjusted for maternal and paternal age (dichotomised as <20 or ≥20 years) and parity, characterized as number of previous pregnancies (0, 1, 2+), as these factors are also associated with increased risk of TS/CT in the ALSPAC sample [16]. We note that parental age and parity may also be associated with greater pregnancy-associated anxiety and depression, with younger and or first-time parents showing increased symptoms due to the novelty of the experience. We also controlled for pre-pregnancy parental psychopathology by examining self-reports of current or past history of severe depression in mothers and fathers and by creating a psychopathology score which comprised reporting a history of any of the following conditions (drug addiction, alcoholism, schizophrenia, anorexia nervosa, severe depression or other psychiatric problems). This score was then grouped into an ordinal variable (0, 1, 2 or more) and added as a dummy variable in the final regression model.

### Analyses

We initially compared simple descriptive statistics for cases and unaffected children. We then used logistic regression analysis to run both unadjusted and adjusted analyses comparing tertiles of anxiety and depression in mother and fathers separately for both TS and TS or CT (TS/CT), though given the greater power we only report the latter outcome. Multivariable models were adjusted for maternal age, socioeconomic status, and parity. We also repeated the analysis with both maternal and paternal mood in the same model to see if one or both were attenuated.

**Fig. 1:**
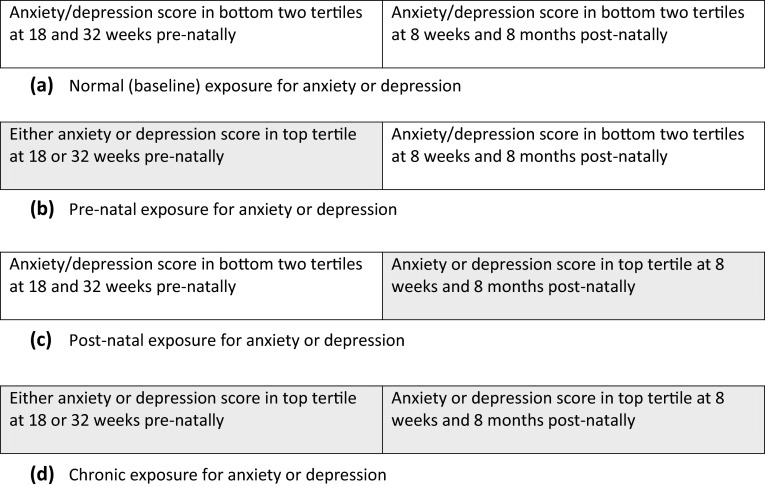
Classification of psychological morbidity in the pre and post-natal periods into a normal, early, late or chronic life course exposure

We then derived a mutually exclusive time-related exposure for both anxiety and depression to compare between a critical/sensitive period exposure or an accumulation “chronic” exposure. Pre-natal exposure to depression or anxiety was defined as being in the top tertile for symptom severity at either 18 or 32 weeks. Post-natal exposure was defined as depression (or anxiety) symptoms in the top tertile at both 8 weeks and 8 months, as high symptom levels at 8 weeks could reflect transitory adverse events related to delivery rather than true psychological morbidity. Each mother or father was therefore classified as belonging to one of four mutually exclusive categories for anxiety as well as for depression: normal (few or no symptoms), pre-natal only (high symptoms prenatally but not post-natally), post-natal (high symptoms post-natally only) or chronic (high symptoms both pre- and post-natally) (see Fig. 1).

## Results

### Univariable analyses

Summary statistics of the unadjusted exposure and confounder variables stratified by the presence or absence of TS/CT demonstrated that mothers of all children regardless of case status had their highest levels of anxiety and depression during pregnancy; the level of anxiety and depression symptoms fell post-natally (Table 1). The same pattern was also seen for paternal anxiety. In case/non-case comparisons, maternal anxiety and depression scores were higher for mothers of children with TS/CT than for mothers of unaffected children at all time points, while only depression at 8 months post-natally appeared worse for fathers of children with TS/CT compared to fathers of unaffected children. Mothers of children with TS/CT were also younger, more likely to be nulliparous and come from poorer areas of residence than mothers of unaffected children.

**Table 1:** Basic descriptive for subjects with Tourette syndrome or chronic tic disorder (TS/CT) and unaffected children according to exposure and confounder status

Variable	TS/CT	Unaffected children	*p* value
	Mean (SD) or %	Mean (SD) or %	
Maternal anxiety total score			
18 weeks pre-natal	5.5 (3.4)	4.6 (3.3)	0.006
32 weeks pre-natal	5.5 (3.4)	4.7 (3.4)	0.02
8 weeks post-natal	4.1 (3.8)	3.1 (3.1)	0.001
8 months post-natal	4.1 (3.8)	3.4 (3.2)	0.03
Maternal depression total score			
18 weeks pre-natal	7.6 (5.0)	6.3 (4.5)	0.003
32 weeks pre-natal	7.3 (5.3)	6.4 (4.8)	0.07
8 weeks post-natal	6.5 (5.3)	5.6 (4.5)	0.03
8 months post-natal	6.5 (5.9)	5.0 (4.5)	0.0006
Paternal anxiety total score			
18 weeks pre-natal	3.0 (2.7)	3.0 (2.7)	0.81
8 weeks post-natal	2.6 (2.4)	2.5 (2.6)	0.81
8 months post-natal	2.3 (1.9)	2.2 (2.4)	0.73
Paternal depression total score			
18 weeks pre-natal	4.4 (3.9)	4.0 (3.7)	0.33
8 weeks post-natal	3.8 (3.4)	3.7 (3.7)	0.63
8 months post-natal	4.1 (3.7)	3.2 (3.5)	0.03
Maternal age			
<20 years (%)	5.0	1.9	0.01
SES factor score			
Most affluent tertile (%)	25.9	36.1	
Middle tertile (%)	24.1	31.9	
Most deprived tertile (%)	50	32.0	<0.0001
Parity (%)			
0	57.8	46.8	
1	30.2	36.4	
2+	12.1	12.1	0.06

### Multivariable analyses of individual time points

Similar results were seen when maternal anxiety was examined in tertiles for unadjusted models, but after adjustment for maternal age, parity and socioeconomic status, only maternal anxiety at 32 weeks gestation remained robustly associated with TS/CT with an odds ratio of 1.82 (95 % CI 1.12, 2.96, *p* = 0.02; trend test across tertiles, *p* = 0.02) (see supplemental Table 1). Conditioning for a past history of maternal depression, if anything strengthened the association (odds ratio 1.94, 95 % CI 1.18, 3.19, *p* = 0.009) while controlling for the psychopathology score showed a slightly weaker association (odds ratio 1.79, 95 % CI 1.09, 2.94, *p* = 0.02). There was no association of TS/CT with paternal anxiety in either the unadjusted or adjusted models. The analogous multivariable results for depression did not support the nominal associations between TS/CT and maternal depression at 18 weeks gestation or with paternal depression at 8 weeks post-natal that were seen in the unadjusted model; instead, the unadjusted associations were sufficiently attenuated after controlling for maternal age, parity and socioeconomic status so that there was no strong evidence for an association between TS/CT and either maternal or paternal depression (see Supplemental Table 2). We also ran models where we mutually adjusted for both maternal and paternal as continuous variables. Conditional on paternal anxiety, maternal anxiety remained predictors of TS/CT at 18 weeks pre-natal and 8 weeks post-natal (*p* values 0.02 and 0.03 respectively) while paternal variables were consistent with chance. For depression, only maternal depression at 18 weeks predicted TS/CT (*p* = 0.02).

### Longitudinal analyses of time period specific or chronic pre-natal/post-natal exposure

#### Parental anxiety

We then examined if exposure within a specific critical/sensitive time period and/or chronic exposure of psychological morbidity across multiple time points showed different associations with TS/CT than those arising from the analyses of isolated time points described above (Table 2). For maternal anxiety, chronic pre- and post-natal exposure was associated with a more than doubling of odds of TS/CT, which was only modestly attenuated in the multivariable model (OR: 2.17, 95 % CI 1.23,3.84, *p* = 0.007). Adjusting for either a pre-pregnancy history of maternal depression or the number of maternal psychological problems did little to alter this result (odds ratios 2.31, 95 % CI 1.29, 4.14 and 2.12, 95 % CI 1.73, 3.83, respectively). In contrast, there was little evidence of any effect on TS/CT risk with paternal anxiety in any period (all *p* values >0.25).

**Table 2:** Association of parental anxiety and depression exposure in the pre-natal, post-natal or both periods with TS and CT

	Unadjusted model			Adjusted model^a^		
	Odds ratio	95 % CI	*p* value	Odds ratio	95 % CI	*p* value
Maternal anxiety						
Normal	1.00			1.00		
Pre-natal	1.39	0.72, 2.68	0.33	1.34	0.68, 2.61	0.40
Post-natal	1.11	0.34, 3.62	0.87	0.79	0.19, 3.33	0.40
Chronic	2.49	1.48, 4.20	<0.001	2.17	1.23, 3.84	0.007
Maternal depression						
Normal	1.00			1.00		
Pre-natal	1.78	0.99, 3.20	0.05	1.86	1.02, 3.39	0.04
Post-natal	0.81	0.19, 3.38	0.75	0.82	0.19, 3.45	0.79
Chronic	1.81	1.05, 3.10	0.03	1.62	0.90, 2.93	0.11
Paternal anxiety						
Normal	1.00			1.00		
Pre-natal	1.37	0.47, 3.91	0.54	1.36	0.47, 3.95	0.58
Post-natal	1.76	0.67, 4.58	0.25	1.71	0.64, 4.52	0.28
Chronic	0.75	0.29, 1.93	0.55	0.76	0.29, 1.99	0.58
Paternal depression						
Normal	1.00			1.00		
Pre-natal	1.16	0.41, 3.33	0.28	1.12	0.38, 3.29	0.84
Post-natal	1.06	0.32, 3.52	0.92	0.97	0.28, 3.30	0.96
Chronic	1.86	0.95, 3.67	0.07	1.87	0.93, 3.75	0.08

^a^Adjusted for maternal or paternal age, socioeconomic status factor (tertiles) and parity

#### Parental depression

There was a modest association signal for maternal pre-natal depression post-adjustment (OR 1.86, 95 % CI 1.02, 3.39, *p* = 0.04) and similar, albeit weaker, effects for chronic depression for both mothers (OR 1.62, 95 % CI 0.90, 2.93, *p* = 0.11) and fathers (OR 1.87, 95 % CI, 0.93, 3.75, *p* = 0.08). Adjustment for a past (pre-pregnancy) history of maternal psychopathology slightly attenuated the association of TS/CT with maternal pre-natal depression (odds ratio 1.78, 95 % CI 0.96, 3.27, *p* = 0.07) though this result was not altered when conditioning on the number of psychological problems. Adjustment for a past (pre-pregnancy) history of paternal psychopathology made little difference to the results, though conditioning on the number of paternal psychological problems further attenuated the associations of chronic paternal depression with TS/CT (odds ratio 1.73, 95 % CI 0.84, 3.54, *p* = 0.14).

## Discussion

This is the first study to examine prospectively the relationship between parental anxiety and depression during the pre- and post-natal period and risk of TS/CT. We found that both maternal anxiety and depression were associated with increased rates of TS/CT in the ALSPAC cohort, although slightly different patterns were seen for each, perhaps indicating different mechanisms of action. In particular, while we initially saw associations between TS/CT and pre-natal maternal anxiety measured at a single time point, this observed effect was actually driven by those mothers with chronic anxiety exposure (both pre- and post-natal) rather than those with anxiety restricted to the pre-natal period. This pattern, in our view, is more suggestive of a genetic rather than an environmental etiology, particularly since heritability analyses in clinical samples indicate that mothers of TS patients may themselves manifest TS carrier status through symptoms of OCD (Hirschtritt/Darrow et al. in submission). For maternal depression, however, we saw a stronger association for pre-natal maternal depression rather than post-natal or chronic depression, suggesting a possible environmental (i.e., non-genetic), time period-specific risk factor, though after adjustment for past maternal depression this was weakened and consistent with chance.

This result is consistent with prior evidence that maternal rather than paternal depression is associated with ADHD risk [17] and the association between maternal anti-depressant use in the first trimester and ADHD [18]. Although we do not know the mechanism of the association between maternal pre-natal depression and offspring TS/CT, one possibility is neuroendocrine effects of depression on the developing fetal brain. There is evidence that maternal stress is associated with down-regulation of placental 11 beta-hydroxysteroid [19] and hence greater exposure to maternal cortisol and possibly dysregulation of offspring cortisol production, though the exact mechanisms are not clear [20, 21]. This could also be associated with maternal anti-depressant use, which we are not able to evaluate directly due to the small amount of medication exposure data available in the ALSPAC cohort and may explain why conditioning for past maternal depression attenuated our association as this may capture medication effects over and above the measure of depression.

We found little evidence for an association between TS/CT and paternal anxiety or depression, though given the high odds ratio (1.86) and associated *p* value of 0.08, it is possible that chronic paternal depression was also associated with increased risk of TS/CT and failed to reach conventional levels of statistical significance due to lack of power (type II error). In addition, fathers did not complete a 32 week questionnaire, the time point which correlated with the strongest maternal effect, so it is possible that there was greater paternal than maternal misclassification of exposure, which would have further attenuated any association.

As noted, there has been very little previous examination of the relationship between parental psychological symptoms and TS/CT in their offspring. We could only identify two previous studies on this topic, both retrospective, and the results were somewhat contradictory [6, 7]. The first study, by Leckman and colleagues, retrospectively examined severity of maternal stress during pregnancy based on life events in 31 TS patients. They found that severe maternal stress predicted tic severity, particularly phonic tic severity. A more recent report by Motlagh and colleagues examined 105 subjects with either TS alone or with TS and comorbid attention deficit hyperactivity disorder (ADHD) recruited through specialty clinics and the Tourette Syndrome Association, 52 children with ADHD alone recruited through specialty clinics, and 65 controls recruited by random digit dialing [7]. Mothers were asked to retrospectively rate family level of stress severity and coping ability, which was dichotomized into a binary variable of severe maternal stress and poor coping. In multivariable models, mothers of children with ADHD had elevated odds ratios for severe stress and poor coping (OR 16.3, 95 % CI 1.5–135, *p* = 0.01). This was also seen for children with TS plus ADHD, although the results were consistent with chance, and were even weaker for children with TS but without ADHD. This association with ADHD is consistent with a previous study from the ALSPAC cohort, although the association was attenuated after adjustment for post-natal symptoms and socioeconomic factors [9]. Taken together, these studies do suggest that there may be an association between TS and maternal psychological stress. However, due to methodological limitations, Motlagh and colleagues could not examine the time course of the putative exposure, adjust for socioeconomic status or examine the role of paternal psychological stress in TS risk.

Thus, this is the first prospective study that has examined both maternal or paternal psychological symptoms and their relationship to TS/CT risk. Not only does this study add to the very small extant literature on potential associations between parental psychological morbidity and TS/CT, the multitude of repeat assessments allows us to test specific life course exposure models. Specifically, had repeat post-natal measures not been available, we would have wrongly concluded that pre-natal anxiety might convey TS/CT risk through a critical exposure period rather than the longitudinal observation that this pre-natal association is driven by mothers who report both pre and post-natal anxiety due to chronicity.

There are several possible explanations that should be considered before assuming that we have observed a causal association. Our associations may reflect a type I error given the multiplicity of statistical tests that have been undertaken. A Bonferroni adjustment would certainly make most of our findings fall within the realms of chance. However, such an approach is regarded by some experts as being overly conservative, and the associations were all stated a priori rather than being a post hoc finding. Equally, despite the large size of the cohort, the number of TS/CT cases was still limited and we were underpowered to detect modest effects, such as odds ratios less than 1.70.

Bias is less of a concern, as exposures were measured prior to case ascertainment and the latter was done blinded to exposure assessment. As in any follow-up study, some of the original sample will have been lost to follow-up. This could have introduced bias if those who were lost to follow-up had the opposite associational pattern (greater anxiety and depression but a lower risk of TS/CT) thereby canceling out the observed effects. While psychological morbidity in the mothers and fathers may well predict “missingness” at a future follow-up, we believe, if anything, this bias is more likely to have resulted in an under-estimate of the true association as it seems more intuitive that such parents with children with TS/CT may be more likely to not attend follow-up assessments rather than the other way around. Given the observational nature of our data, we have attempted to adjust for confounding, but accept that due to inadequate adjustment, there may still be residual confounding. However, it has been argued [22] that in the case of residual confounding, similar associations should be seen with paternal as well as maternal exposure, which was not the case for our anxiety results, and which were only modestly attenuated in the multivariable models. Reverse causation is not a possibility in this study as the pregnancy and 8 months exposures could not have been influenced secondary to TS/CT in the offspring. Finally, we need to consider causal explanations such that there is broader sub-clinical (“forme fruste”) phenotype of TS/CT that may include anxiety and depression as part of the symptom spectrum. In this case, the associations we have observed would be due to a shared genetic rather than a specific neurodevelopmental causal pathway. Alternatively pre-natal maternal depression may influence offspring risk of TS/CT through epigenetic mechanisms or secondary to drug toxicity from pharmacological treatment of the mother.

## Conclusions

Chronic maternal anxiety and pre-natal depression appear to be associated with subsequent risk of CT and TS though the latter may be due to a past history of maternal depression. Further research needs to replicate these findings and try to determine whether these associations are part of a broader CT/TS phenotype or reflect specific etiological mechanisms operating through genetic, epigenetic or environmental pathways.

## Electronic supplementary material

Below is the link to the electronic supplementary material.

Supplementary material 1 (DOCX 18 kb)
