# Supplementary material for: Parental mood during pregnancy and post-natally is associated with offspring risk of Tourette syndrome or chronic tics: prospective data from the Avon Longitudinal Study of Parents and Children (ALSPAC)
Source: Eur Child Adolesc Psychiatry. 2015 Jul 15;25:373–81. doi: 10.1007/s00787-015-0742-0 (PMC4820468; doi:10.1007/s00787-015-0742-0)
Supplement: Supplementary file 1 — Supplementary material 1 (DOCX 18 kb) [file 787_2015_742_MOESM1_ESM.docx]

| Maternal reported anxiety | | Unadjusted model | | | |  | Adjusted model** | | |
| --- | --- | --- | --- | --- | --- | --- | --- | --- | --- |
| Time period | | Odds ratio* | | 95% CI | p-value |  | Odds ratio* | 95% CI | p-value |
| 18 weeks prenatal |  |  | |  |  |  |  |  |  |
| Tertile 2 | | 1.66 | | 1.04, 2.65 | 0.03 |  | 1.53 | 0.94, 2.50 | 0.09 |
| Tertile 3 |  | 1.74 | | 1.07, 2.82 | 0.03 |  | 1.45 | 0.86, 2.43 | 0.17 |
| p-value for trend | | | |  | 0.02 |  |  |  | 0.14 |
| 32 weeks prenatal | |  | |  |  |  |  |  |  |
| Tertile 2 | | 1.08 | | 0.67, 1.74 | 0.74 |  | 1.24 | 0.75, 2.05 | 0.41 |
| Tertile 3 |  | 1.79 | | 1.15, 2.79 | 0.01 |  | 1.82 | 1.12, 2.96 | 0.02 |
| p-value for trend | | | |  | 0.01 |  |  |  | 0.02 |
| 8 weeks post-natal | |  | |  |  |  |  |  |  |
| Tertile 2 |  | | 1.01 | 0.63, 1.63 | 0.96 |  | 0.92 | 0.56, 1.50 | 0.73 |
| Tertile 3 |  | | 1.77 | 1.12, 2.79 | 0.02 |  | 1.42 | 0.88, 2.31 | 0.15 |
| p-value for trend | | | |  | 0.02 |  |  |  | 0.17 |
| 8 months post-natal | |  | |  |  |  |  |  |  |
| Tertile 2 | | 1.32 | | 0.81, 2.16 | 0.26 |  | 1.20 | 0.71, 2.01 | 0.50 |
| Tertile 3 |  | 1.38 | | 0.89, 2.13 | 0.15 |  | 1.23 | 0.78, 1.96 | 0.38 |
| p-value for trend | | | |  | 0.12 |  |  |  | 0.36 |
|  |  |  | |  |  |  |  |  |  |
| Paternal reported anxiety | | | |  |  |  |  |  |  |
| Time period |  |  | |  |  |  |  |  |  |
| 18 weeks prenatal |  |  | |  |  |  |  |  |  |
| Tertile 2 | | 1.09 | | 0.67, 1.77 | 0.72 |  | 1.06 | 0.60, 1.86 | 0.84 |
| Tertile 3 |  | 1.14 | | 0.67, 1.94 | 0.62 |  | 1.25 | 0.69, 2.28 | 0.46 |
| p-value for trend | | | |  | 0.50 |  |  |  | 0.47 |
| post-natal | |  | |  |  |  |  |  |  |
| 8 weeks post-natal | | | |  |  |  |  |  |  |
| Tertile 2 |  | 1.44 | | 0.87, 2.39 | 0.16 |  | 1.28 | 0.74, 2.22 | 0.37 |
| Tertile 3 |  | 1.25 | | 0.74, 2.11 | 0.41 |  | 1.16 | 0.66, 2.04 | 0.60 |
| p-value for trend | | | |  | 0.35 |  |  |  | 0.55 |
| 8 months post-natal | |  | |  |  |  |  |  |  |
| Tertile 2 | | 1.57 | | 0.85, 2.91 | 0.16 |  | 1.51 | 0.78, 2.94 | 0.22 |
| Tertile 3 |  | 1.46 | | 0.84, 2.51 | 0.18 |  | 1.22 | 0.66, 2.23 | 0.53 |
| p-value for trend | | | |  | 0.16 |  |  |  | 0.47 |
| * odds ratios to baseline tertile; ** adjusted for maternal or paternal age, socio-economic status factor (tertiles) and parity  Supplemental table 1: Associations between maternal and paternal reported anxiety with Tourette syndrome or chronic tics or controls with and without adjustment for confounders. | | | | | | | | | |

|  | | Unadjusted model | | |  | Adjusted model** | | |
| --- | --- | --- | --- | --- | --- | --- | --- | --- |
| Time period | | Odds ratio | 95% CI | p-value |  | Odds ratio | 95% CI | p-value |
| Maternal reported depression | | |  |  |  |  |  |  |
| 18 weeks prenatal |  |  |  |  |  |  |  |  |
| Tertile 2 | | 0.90 | 0.54, 1.51 | 0.7 |  | 0.77 | 0.44, 1.32 | 0.34 |
| Tertile 3 |  | 1.77 | 1.14, 2.76 | 0.01 |  | 1.47 | 0.91, 2.35 | 0.11 |
| p-value for trend | | |  | 0.01 |  |  |  | 0.12 |
| 32 weeks prenatal | |  |  |  |  |  |  |  |
| Tertile 2 | | 1.42 | 0.89, 2.27 | 0.15 |  | 1.48 | 0.91, 2.42 | 0.11 |
| Tertile 3 |  | 1.47 | 0.93, 2.31 | 0.10 |  | 1.23 | 0.74, 2.02 | 0.42 |
| p-value for trend | | |  | 0.09 |  |  |  | 0.40 |
| 8 weeks post-natal | |  |  |  |  |  |  |  |
| Tertile 2 |  | 1.49 | 0.95, 2.35 | 0.08 |  | 1.47 | 0.92, 2.35 | 0.10 |
| Tertile 3 |  | 1.31 | 0.82, 2.09 | 0.26 |  | 1.08 | 0.65, 1.80 | 0.76 |
| p-value for trend | | |  | 0.25 |  |  |  | 0.70 |
| 8 months post-natal | |  |  |  |  |  |  |  |
| Tertile 2 | | 1.34 | 0.83, 2.16 | 0.23 |  | 1.31 | 0.80, 2.16 | 0.29 |
| Tertile 3 |  | 1.54 | 0.96, 2.48 | 0.08 |  | 1.42 | 0.86, 2.36 | 0.17 |
| p-value for trend | | |  | 0.08 |  |  |  | 0.17 |
|  |  |  |  |  |  |  |  |  |
| Paternal reported depression | | |  |  |  |  |  |  |
| 18 weeks prenatal |  |  |  |  |  |  |  |  |
| Tertile 2 | | 1.14 | 0.68, 1.90 | 0.62 |  | 1.09 | 0.60, 2.00 | 0.78 |
| Tertile 3 |  | 1.40 | 0.87, 2.25 | 0.17 |  | 1.57 | 0.91, 2.70 | 0.11 |
| p-value for trend | | |  | 0.17 |  |  |  | 0.12 |
| 8 weeks post-natal | |  |  |  |  |  |  |  |
| Tertile 2 |  | 1.53 | 0.90, 2.60 | 0.12 |  | 1.27 | 0.72, 2.24 | 0.40 |
| Tertile 3 |  | 1.34 | 0.78, 2.31 | 0.29 |  | 1.18 | 0.67, 2.10 | 0.57 |
| p-value for trend | | |  | 0.30 |  |  |  | 0.57 |
| 8 months post-natal | |  |  |  |  |  |  |  |
| Tertile 2 | | 0.87 | 0.46, 1.66 | 0.67 |  | 0.8 | 0.40, 1.60 | 0.54 |
| Tertile 3 |  | 1.87 | 1.09, 3.20 | 0.02 |  | 1.54 | 0.85, 2.78 | 0.15 |
| p-value for trend | | |  | 0.03 |  |  |  | 0.18 |
|  |  |  |  |  |  |  |  |  |
| * odds ratios compared to baseline tertile | | | | |  |  |  |  |
| ** adjusted for maternal or paternal age, socio-economic status factor (tertiles) and parity | | | | | | | | |

Supplemental table 2: Associations between maternal and paternal reported depression with Tourette syndrome or chronic tics or controls with and without adjustment for confounders.
